# Spatial mosaic evolution of snail defensive traits

**DOI:** 10.1186/1471-2148-7-50

**Published:** 2007-03-30

**Authors:** Steven G Johnson, C Darrin Hulsey, Francisco J García de León

**Affiliations:** 1 Department of Biological Sciences, University of New Orleans, 2000 Lake Shore Drive, New Orleans, LA, 70148 USA; 2 Department of Biology, Georgia Tech, 310 Ferst Drive, Atlanta, Georgia, 30332, USA; 3 Centro de Investigaciones Biologicas del Noroeste, P.O. Box 128, La Paz, B.C.S. Mexico

## Abstract

**Background:**

Recent models suggest that escalating reciprocal selection among antagonistically interacting species is predicted to occur in areas of higher resource productivity. In a putatively coevolved interaction between a freshwater snail (*Mexipyrgus churinceanus*) and a molluscivorous cichlid (*Herichthys minckleyi*), we examined three components of this interaction: 1) spatial variation in two putative defensive traits, crushing resistance and shell pigmentation; 2) whether abiotic variables or frequency of molariform cichlids are associated with spatial patterns of crushing resistance and shell pigmentation and 3) whether variation in primary productivity accounted for small-scale variation in these defensive traits.

**Results:**

Using spatial autocorrelation to account for genetic and geographic divergence among populations, we found no autocorrelation among populations at small geographic and genetic distances for the two defensive traits. There was also no correlation between abiotic variables (temperature and conductivity) and snail defensive traits. However, crushing resistance and frequency of pigmented shells were negatively correlated with molariform frequency. Crushing resistance and levels of pigmentation were significantly higher in habitats dominated by aquatic macrophytes, and both traits are phenotypically correlated.

**Conclusion:**

Crushing resistance and pigmentation of *M. churinceanus *exhibit striking variation at small spatial scales often associated with differences in primary productivity, substrate coloration and the frequency of molariform cichlids. These local geographic differences may result from among-habitat variation in how resource productivity interacts to promote escalation in prey defenses.

## Background

Spatial variation in defensive traits is common in many antagonistic interactions [1-3]. The geographic mosaic theory of coevolution suggests that variation in ecological factors may underlie this patchiness in the phenotypic outcome of interactions, resulting in cold and hotspots of coevolution [2,4,5]. Despite evidence for coevolutionary cold and hotspots, very little evidence exists on how ecological conditions may enhance or constrain coevolution between antagonists. One current model suggests that coevolution might be related to gradients in resource productivity [6], with hotspots of coevolutionary selection associated with areas of high prey abundance. In specialist predator-prey interactions, predators have the greatest influence on prey phenotypes in geographic areas where the prey is most abundant whereas the predator exerts weaker selection in areas where prey are less abundant. Conditions of high prey abundance can occur when the abiotic environment is most favorable for prey metabolism or prey resources are of high quality/abundance. In the current study, we examine the effect of abiotic and biotic factors on spatial variation in the evolution of putative defensive traits of the freshwater snail *Mexipyrgus churinceanus*.

**Figure 1:**
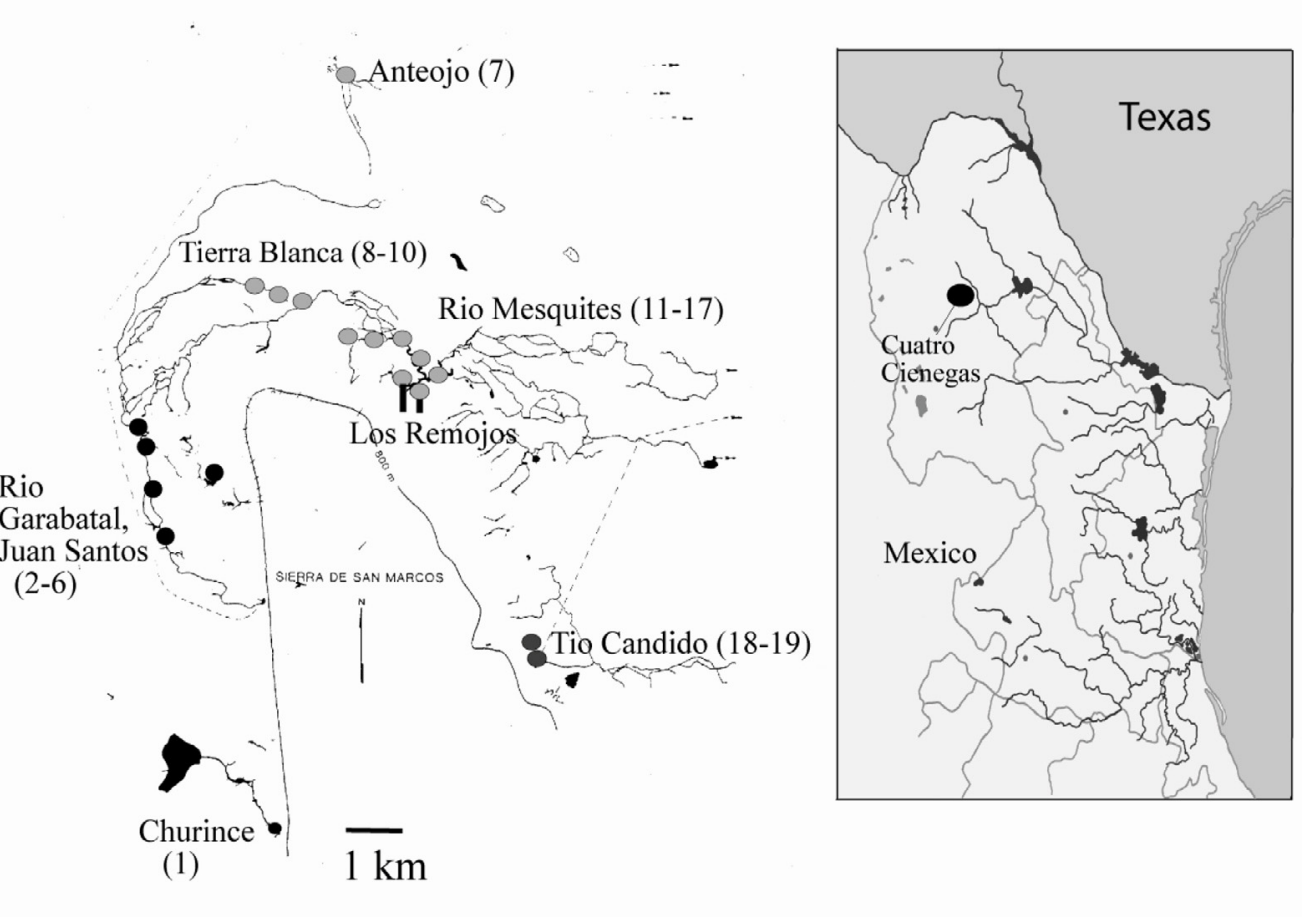
Collecting sites of *Mexipyrgus churinceanus *from spring-fed habitats in the Cuatro Ciénegas valley located in the center of the Chihuahuan desert of northeastern Mexico. These pools and streams are arrayed around the Sierra de San Marcos that bisects the center of the valley. Population numbers are shown on the map and referred to in Table 1.

**Figure 2:**
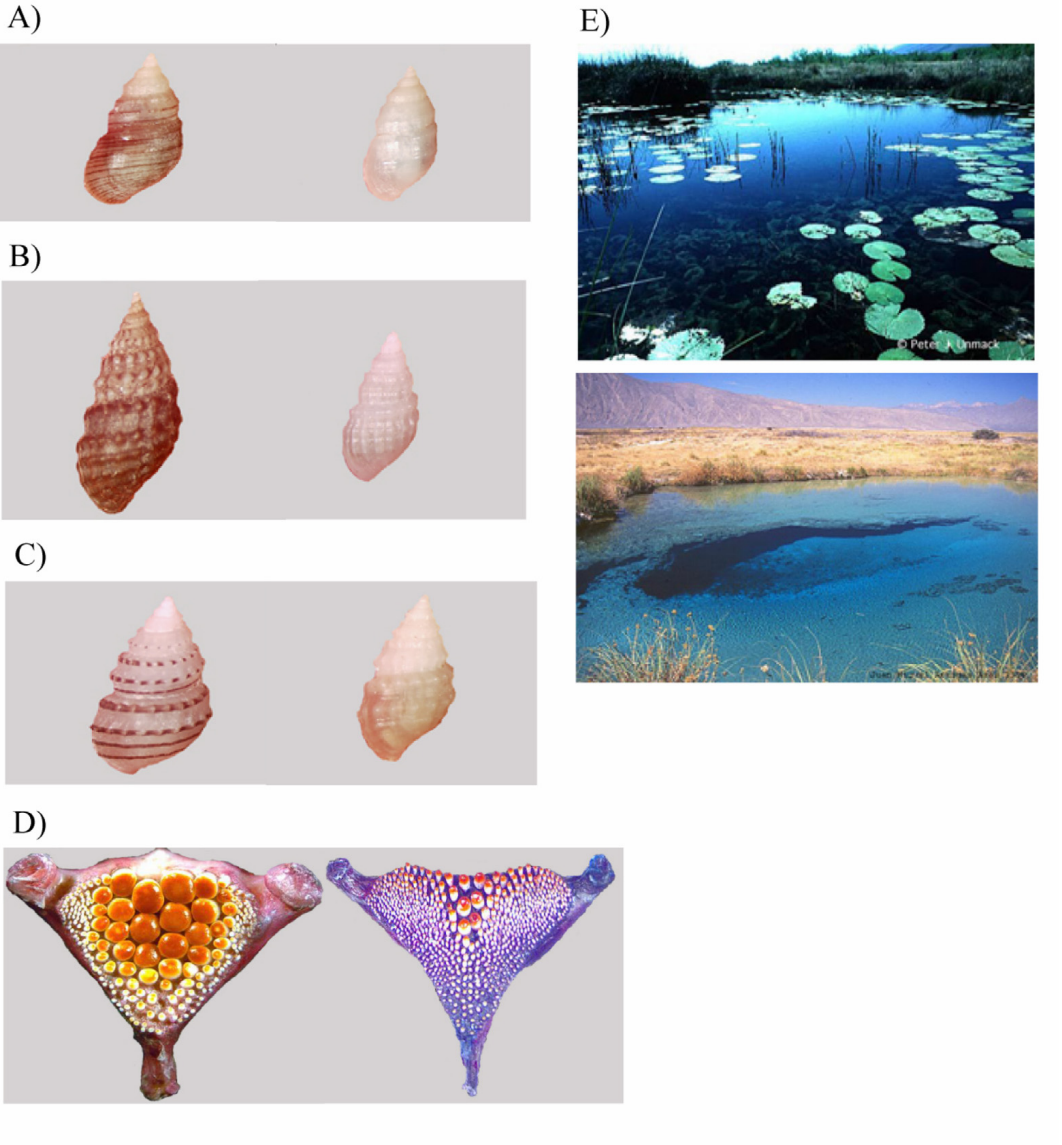
Representative images (A-C) of the hydrobiid snail, *Mexipyrgus churinceanus*, collected from nearby populations to illustrate small scale variation in size and shell pigmentation. *Herichthys minckleyi *papilliforms (right figure D) exhibit gill arches modified into more gracile pharyngeal jaws with small muscles and pointed teeth that are ineffective at crushing snails while *H. minckleyi *molariforms (left figure D) have robust muscles and enlarged crushing teeth on their pharyngeal jaws that seem clearly modified to crush snails. Figure E portrays heterogeneity in habitats with *Nymphaea *(top) and without *Nymphaea *(bottom).

*Mexipyrgus churinceanus *as well as its polymorphic fish predator *Herichthys minckleyi *are endemic to the isolated Cuatro Ciénegas valley in the Mexican Chihuahuan desert (Fig. 1) and exhibit putatively co-evolved phenotypes [7-9]. These phenotypes may currently be coevolving because *Mexipyrgus *exhibits striking examples of morphological differentiation in shell morphology and pigmentation among habitats [[10-12]; Fig. 2]. *Herichthys minckleyi *is also unusual because this cichlid fish exhibits two alternative pharyngeal jaw morphologies [[13,14]; Fig. 2]. In "papilliform" *H. minckleyi*, the gill arches are modified into a gracile pharyngeal jaw and this morph is specialized to shred aquatic plants and detritus [15]. Alternatively, "molariforms" possess enlarged crushing molariform teeth and robust pharyngeal muscles [16] and are extremely proficient at crushing *M. churinceanus *[15]. Molariform cichlids are the primary snail-crushing predator in Cuatro Ciénegas.

Given considerable spatial variation in shell pigmentation and morphology, we hypothesize that several types of snail defenses have evolved in response to predation by molariform *H. minckleyi*. For instance, cryptic coloration against different benthic substrates may minimize detection by molariform cichlids. The abundance of emergent aquatic vegetation, such as *Nymphaea *water lilies, is highly variable in these aquatic habitats, and may be primarily responsible for the coloration of the substrate. Darker substrates dominate in areas with dense *Nymphaea *stands and light, marl-colored substrates occur in the absence of *Nymphaea *(Fig. 2). Unpigmented *M. churinceanus *may be more cryptic and common in habitats without *Nymphaea *whereas, in heavily vegetated habitats, snails with extensive shell pigmentation may dominate because they effectively match these darker substrates.

Shell pigmentation is not the only defense *M. churinceanus *may exhibit in response to molariform predation. Crushing resistance is likely the most functionally important component of shell defense against molariform *H. minckleyi *[15] because it allows snails to resist predation once detected. Because previous studies of defensive traits in gastropods indicate that shell size and shape both influence crushing resistance [17,18], we first examine whether size or shape is a better predictor of variation in crushing resistance. Next, we assess potential causes of spatial divergence in *Mexipyrgus *defensive traits. The abiotic characteristics of Cuatro Ciénegas may be unusually favorable for the production of elaborate snail shells. The spring-fed habitats are geothermally heated and high in dissolved minerals [7]. For mollusks, higher temperatures permit elaboration of their shells because calcium carbonate is less soluble in warmer water [19,20]. Maintaining robust shells is also physiologically cheap in constant aquatic environments with elevated mineral content [20,21] especially since Ca++ availability is correlated with the availability of other dissolved minerals in Cuatro Ciénegas [22]. We examined whether spatial variation in temperature and conductivity are positively correlated with snail defensive traits.

Alternatively, variation in shell strength might be primarily a response to biotic influences. We address whether spatial variation in snail defenses is associated with two biotic factors: molariform frequency and resource productivity. If molariform *H. minckleyi *predation is the primary force driving spatial variation in snail defensive traits, we might predict that snail populations with higher frequencies of molariforms should exhibit greater crushing resistance and pigmentation. We also assess whether habitats with greater primary productivity promote escalation of defensive snail phenotypes. We determine whether, in habitats with greater abundance of *Nymphaea, Mexipyrgus *exhibits more shell pigmentation and crushing resistance. For *Mexipyrgus*, variation in resource productivity is likely to be driven by the abundance of *Nymphaea *because in habitats with greater primary productivity, there is a greater abundance of bacteria and fungi in the soft substrates in which *Mexipyrgus *feeds (Johnson, unpublished results). Increased resource availability probably allows greater investment in costly shell material, leading to the prediction that hotspots may occur in areas with greater food resources for snails.

A critical assumption of these population-based measures of phenotypic divergence is that they are statistically independent. This assumption may be violated because nearby snail populations may experience similar predation pressure because cichlids are more mobile than these brooding snails. To address this issue we assess whether the similarity of shell defensive traits among nearby populations can be attributed to either geographic proximity or genetic similarity [23-25]. Genealogical relationships within species based on geographic and genetic distances among populations can be used in conjunction with spatial autocorrelation approaches to determine whether defensive structures of nearby populations are positively autocorrelated. A lack of spatial autocorrelation suggests that defenses are distributed in a mosaic-like fashion across the landscape. If phenotypic divergence in crushing resistance and pigmentation is the result of predator-prey interactions with *H. minckleyi*, and this divergence occurs at very small spatial scales in a geographically mosaic fashion, we predict there will be no significant positive autocorrelation at small spatial and genetic distances. In the current study, we determine genetic distances between snail populations from a previously published study of mtDNA sequence variation in *Mexipyrgus *[26].

We address three questions concerning the evolution of defenses in *Mexipyrgus*. First, we examine spatial variation in two putative defensive traits, crushing resistance and shell pigmentation. Then, we tested whether abiotic or biotic variables account for spatial patterns of crushing resistance and shell pigmentation. Finally, we determine whether variation in primary productivity accounts for small-scale variation in these defensive traits.

## Results

### Spatial Variation of Shell Crushing Resistance and Pigmentation

**Figure 3:**
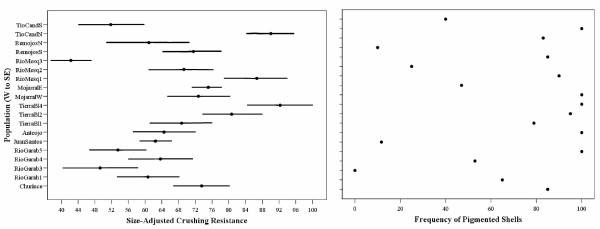
**A) **Average size-adjusted crushing resistance (95% Confidence Intervals) for *M. churinceanus *populations. **B) **Frequency of banded snails for *M. churinceanus *populations. Populations along the Y-axis are arranged on a transect from southwestern populations to the northern Rio Mesquite down to the southeastern lobe.

**Figure 4:**
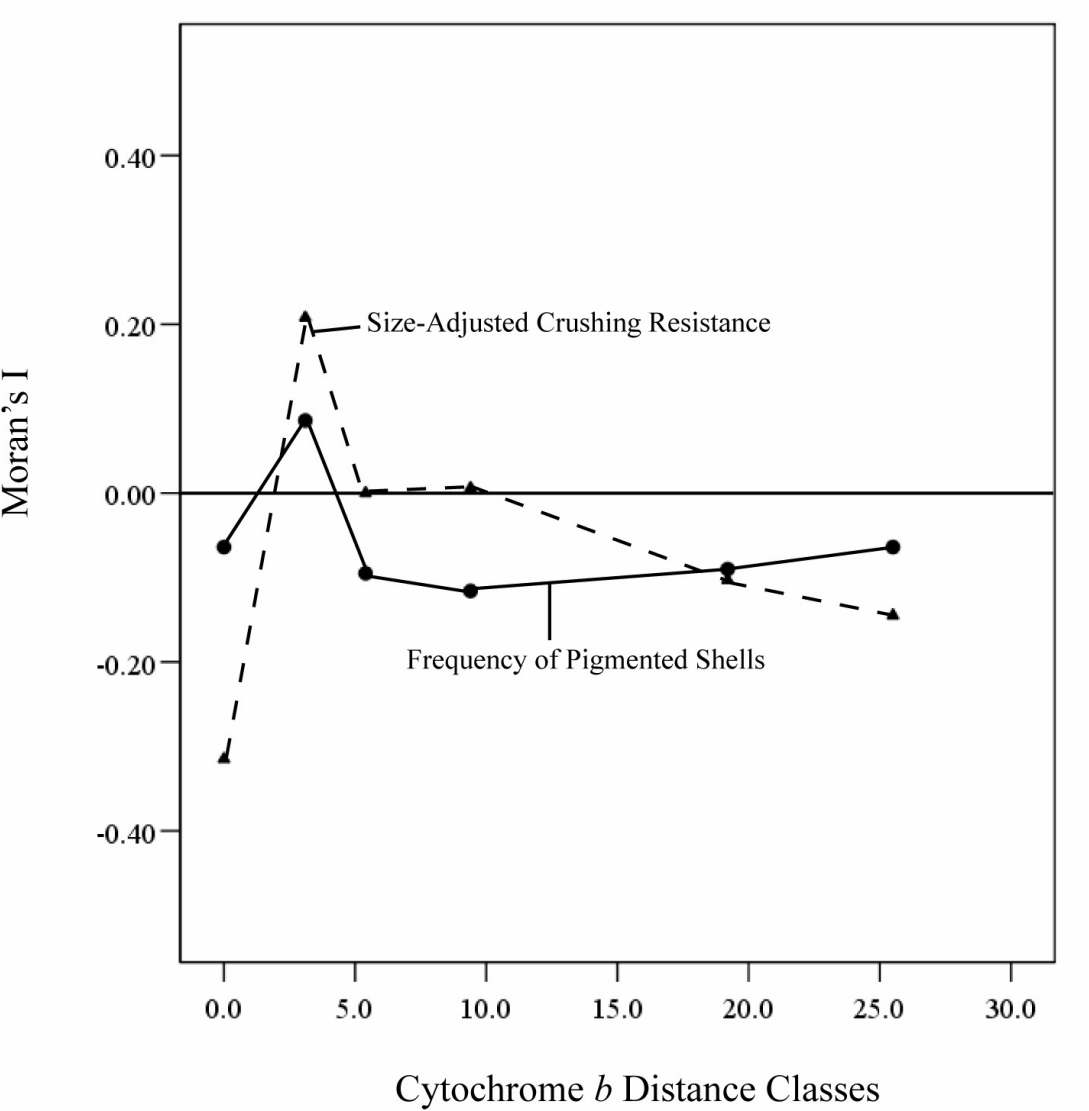
**A and B **Relationship between Moran's I and cytochrome *b *pairwise distance classes (correlograms) for two *Mexipyrgus *traits: size-adjusted crushing resistance (triangles and dashed line) and frequency of pigmented shells (closed circles and solid line).

Mean and standard errors of crushing resistance and shell size are presented in Table 1. The five linear morphological variables had high positive loadings (> 0.96) on the first principal component, and over 94% of the total variance was explained by this first PC, which we interpret as a measure of overall shell size. Overall shell size (PC1 scores) showed considerable variation among populations (Table 1). Covariance PCA analysis revealed one major shape component representing a contrast between aperture size and spire length. This shape component explained about 5% of the total variance, and individuals with positive scores for this component have relatively large apertures and shortened spires. Multiple regression analysis of crushing resistance revealed that shell size and shape were both significantly positively related to crushing resistance. Shell size explained most of the variation in crushing resistance (standardized β = 0.662, df = 1, 503; R^2 ^= 43.9%, p < 0.0001), whereas shell shape explained little variation in crushing resistance (standardized β = 0.077, df = 2, 503; R^2 ^= 0.006%, p = 0.02). We next used analysis of covariance to examine the effect of population on crushing resistance using size as a covariate. The interaction term (shell size by population) was not significant (p = 0.24), and there was a highly significant effect of population on size-adjusted crushing resistance (F = 6.3, df = 18, 467, p < 0.001). There was considerable spatial variation in size-adjusted crushing resistance, ranging from 42.3 Newtons in a Rio Mesquites population to 92.3 Newtons in a Tierra Blanca population (Fig. 3a). The correlograms of size-adjusted crushing resistance and all distance measures were not significant (see Fig. 4 for correlogram using cytochrome *b *distance), indicating the absence of autocorrelation among populations at any scale.

**Table 1:** Mean (95% CI) size-adjusted crushing resistance (SACR), mean PC1 scores (± 2 SE), molariform frequency (MF), and mean number of bands (NB) for 19 *Mexipyrgus churinceanus *populations.

Drainage/Population				
**Western**	SACR	PC1	MF	NB
1. Laguna Churince (n = 20)	73.5(66.0, 81.0)	0.47(0.13)	53.36	9.7(1.2)
2. Rio Garabatal 1 (n = 20)	60.7(53.2, 68.1)	-0.40(0.09)		3.5(0.7)
3. Rio Garabatal 3 (n = 15)	49.2(40.7, 57.8)	0.27(0.10)		0
4. Rio Garabatal 4 (n = 17)	63.7(55.7, 71.7)	0.16(0.14)		3.9(1.0)
5. Rio Garabatal 5 (n = 20)	53.5(46.1, 60.9)	0.05(0.10)		6.7(0.6)
6. Juan Santos (n = 60)	62.47(58.2, 66.7)	0.01(0.05)	77.5	9.7(1.2)
**Rio Mesquites**				
7. Anteojo (n = 19)	64.5(56.9, 72.2)	-0.39(0.13)		13.6(1.0)
8. Tierra Blanca 1 (n = 19)	68.7(61.0, 76.5)	0.67(0.14)		14.2(1.8)
9. Tierra Blanca 2 (n = 20)	80.7(73.1, 88.2)	0.62(0.10)		15.7(1.4)
10. Tierra Blanca 4 (n = 20)	92.3(84.1, 100.4)	1.32(0.06)	31.03	17.3(1.3)
11. Mojarral West (n = 40)	72.7(65.0, 80.4)	-2.14(0.05)	49.77	2.6(1.2)
12. Mojarral East (n = 83)	75.14(71.3, 78.9)	-0.37(0.11)	44.01	4.5(0.9)
13. Rio Mesquites 1 (n = 20)	86.7(78.9, 94.5)	0.94(0.09)	57.75	9.8(1.2)
14. Rio Mesquites 2 (n = 20)	69.3(61.6, 76.9)	0.76(0.12)		2.4(1.0)
15. Rio Mesquites 3 (n = 20)	42.3(34.8, 49.7)	0.38(0.07)		5.1(1.2)
16. Los RemojosN (n = 12)	60.9(51.3, 70.5)	0.29(0.09)	40.00	10.3(2.1)
17. Los RemojosS (n = 20)	71.5(64.2, 78.9)	-0.14(0.07)	62.00	1.6(1.1)
**Southeastern**				
18. Tio CandidoN (n = 40)	90.0(84.2, 95.9)	0.96(0.05)	22.00	5.6(0.2)
19. Tio CandidoS (n = 20)	51.8(44.0, 59.5)	-0.94(0.08)	63.86	0.9(0.2)

There was considerable variation in the frequency of pigmentation among populations (Fig. 3b) and there was a significant association between frequency of pigmentation and population of origin (likelihood ratio χ^2 ^= 310.7, n = 19, p < 0.001). The correlograms of pigmentation frequency and all distance measures were not significant (see figure 4 for correlogram using cytochrome *b *distance classes), again indicating the absence of autocorrelation among populations at any scale.

### Abiotic and Biotic Influences on Crushing Resistance and Pigmentation

**Figure 5:**
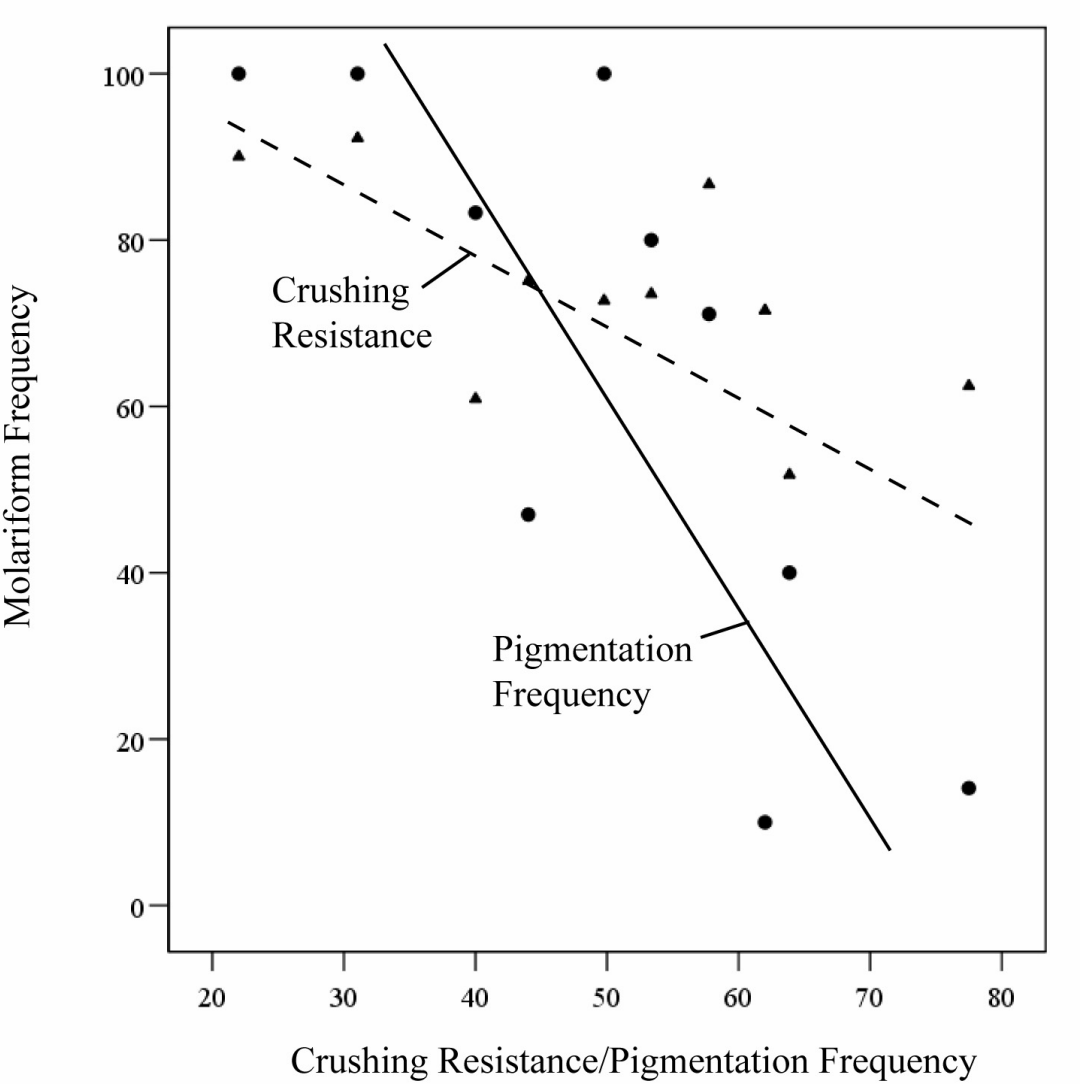
Relationship between frequency of molariform cichlids and both adjusted marginal mean crushing resistance (triangles and dashed line) and frequency of pigmented shells (circles and solid line).

There was no significant correlation between size-adjusted crushing and temperature (r = 0.32, n = 19, *p *= 0.19) or conductivity (r = -0.40, n = 19, *p *= 0.09). Similarly, there was no significant correlation between pigmentation frequency and temperature (r = -0.01, n = 19, *p *= 0.17) or conductivity (r = -0.23, n = 19, *p *= 0.13). In contrast, there were significant negative correlations between molariform frequency and both size-adjusted crushing resistance (r = -0.63, n = 10, *p *= 0.05; Fig. 5) and average frequency of pigmentation (r = -0.78, n = 10, *p *< 0.01; Fig. 5). Using mean population estimates, we examined the correlation between pigmentation patterns and crushing resistance. There was a highly significant positive correlation between number of bands and crushing resistance (r = 0.58, n = 19, *p *< 0.01).

In the two of the three paired adjacent populations that differ in *Nymphaea *abundance (RM1/RM3 and Tio Candido S/N), crushing resistance was higher where *Nymphaea *was abundant (Figure 3a). For example, Tio Candido N had significantly higher size-adjusted crushing resistance than Tio Candido S (90.0 and 51.8, respectively; F = 20.82, df = 1, 58, *p *< 0.001). Similarly, RM1 had significantly higher crushing resistance than nearby RM3 (86.7 and 42.3, respectively; F = 58.62, df = 1, 38; *p *< 0.001). The Los Remojos populations did not differ significantly in size-adjusted crushing resistance (F = 0.12; df = 1, 30, *p *> 0.5). In the paired adjacent populations, the frequency of pigmented shells was significantly higher in darker versus lighter substrates in all 3 comparisons: Rio Mesquites 1 and 3 (90% versus 25%, likelihood ratio = 19.1, d.f. = 1, *p *< 0.001); Los Remojos N and S (83.3% versus 10%, likelihood ratio = 18.5, d.f. = 1, *p *< 0.001); and Tio Candido N and S (100% versus 40%, likelihood ratio = 33.1, d.f. = 1, *p *< 0.001).

## Discussion

Pronounced geographic differences in prey defenses are probably common [5,27]. While the unusually high crushing resistance of *Mexipyrgus churinceanus *in Cuatro Ciénegas and the presence of the molariform cichlids are suggestive of coevolutionary selection [8,15], the current study provides three important insights. First, there is considerable small-scale spatial variation in crushing resistance and pigmentation that exhibits a mosaic distribution with no significant genetic or spatial autocorrelation. Also, there was a significant negative correlation between the frequency of molariforms and both size-adjusted crushing resistance and frequency of shell pigmentation, but no relationship between these defensive traits and the abiotic variables. Lastly, crushing resistance and pigmentation are significantly higher in habitats dominated by aquatic macrophytes.

To address whether prey defenses have evolved independently across the geographic range of *Mexipyrgus churinceanus *requires an assessment of phenotypic variation and the spatial autocorrelation of these traits among populations at small genetic and geographic distances. There is significant spatial variation in *M. churinceanus *crushing resistance and pigmentation at small spatial scales suggesting that certain factors may cause this mosaic distribution of snail defensive traits. In paired populations separated by very small linear and genetic distances, elevated crushing resistance occurs in habitats containing extensive *Nymphaea *stands. Crushing resistance is dramatically lower in light colored substrates lacking *Nymphaea*. Because Tio Candido and Rio Mesquites populations represent distinct lineages based on mtDNA sequence differentiation [26], it suggests that these represent convergent patterns of increased crushing resistance and tantalizingly suggests these differences may be driven by resource availability. In habitats with greater primary productivity, there is probably greater abundance of bacteria and fungi upon which *Mexipyrgus *feeds extensively (Johnson, unpublished results). Increased resource availability likely allows greater investment in costly shell material, and experiments that manipulate resource availability to test its effect on *Mexipyrgus *shell strength would provide a further test of this hypothesis.

Although there is considerable documentation of hotspots and coldspots in coevolved antagonistic interactions, how spatial processes and community composition promote coevolution and generate selection mosaics has received less attention. A recent study of herbivorous weevils and their *Camellia *host plant indicate that escalation in armaments only occurred in southern latitudes [3]. More northern host populations had reduced resistance but exploitation and damage by weevils was even greater in these populations, suggesting that some unknown ecological factor may constrain resistance in northern host populations. There has been speculation that the exaggerated shell form of *Mexipyrgus *is the result of coevolutionary interactions between endemic snails and the molluscivorous morph of *H. minckleyi *[8]. Surprisingly, there was a highly significant negative relationship between molariform frequency and size-adjusted crushing resistance among ten populations, suggesting that molariform fitness declines as snail crushing resistance increases. Molariform cichlids may have a greater fitness advantage in resource-poor environments where snail crushing resistance is lower, and the limited availability of plant material reduces papilliform fitness. In *Nymphaea *habitats, increased snail crushing resistance may reduce molariform fitness due to increased costs of crushing, while papilliform fitness is higher due to increased availability of plant material for shredding. We plan to experimentally test whether these alternative cichlid morphs have fitness trade-offs in different resource environments, and more quantitatively measure resources available to *Mexipyrgus*.

Microspatial variation in *Mexipyrgus *shell pigmentation is also associated with different substrate coloration and there is no evidence of genetic or spatial autocorrelation among populations. In the three geographically-paired populations, snails with pigmented bands were significantly more common in *Nymphaea *habitats and unbanded snails were more common where the benthic substrate was light and marled- colored. Unbanded snails are probably more cryptic against lighter benthic substrates and banded snails are more cryptic against the darker substrates found in association with *Nymphaea*. This type of background matching by prey under selection from highly visual predators is common in many organisms [28-30] and experimental evidence that banding patterns are cryptic against different substrate backgrounds would be an interesting line of further research.

The positive relationship between the two snail traits across environments suggest that pigmentation and crushing resistance may represent phenotypically-correlated traits. In resource-poor environments where *Nymphaea *is absent, investment in crushing resistance may be constrained due to resource limitation, so that crypsis is the most effective defense against molariform predation. We suspect that investment in shell pigmentation is not as costly as having more robust shells, and that snails are capable of inexpensively modifying their pigmentation in *Nymphaea *habitats to increase crypsis. Whether correlational selection acts on pigmentation patterns and shell strength in these habitats requires a deeper understanding of the phenotypic plasticity in both traits. One hypothesis is that increased pigmentation in these environments does not increase background matching, and pigmentation only increases due to correlational selection on crushing resistance. This hypothesis could be easily refuted if predation success on banded shells is less in *Nymphaea *habitats. Given evidence for inducible defenses in snails [31-33], we also need to assess the heritability and phenotypic plasticity of crushing resistance and pigmentation.

## Conclusion

Spatial variation in two prey defense traits, crushing resistance and shell pigmentation, exhibits striking variation at small spatial scales often associated with habitat differences in primary productivity and substrate coloration. These local geographic differences may result from among-habitat variation in how resource productivity interacts to promote escalation in prey defenses. Because molariform *H. minckleyi *undoubtedly use their jaws to crush snails, Cuatro Ciénegas may represent an ideal system for investigating mosaic predator-prey coevolution. In future studies, we plan to examine local adaptation of molariforms, escalation of molariform traits in resource rich environments, and heritability and phenotypic plasticity of both crushing resistance and pigmentation across resource gradients.

## Methods

### Spatial Variation and Autocorrelation of Shell Crushing Resistance and Pigmentation

We sampled nineteen sites containing *Mexipyrgus churinceanus *from various drainages throughout the entire Cuatro Ciénegas basin (Fig. 1; Table 1). Five linear shell measurements (shell length, shell width, spire height, and aperture length and width) commonly used in gastropod studies [25,31,34] were measured using Image-Pro Express. All morphological traits were log10-transformed prior to analysis. Crushing resistance of adult *Mexipyrgus churinceanus *was measured in 19 populations. The snails were placed in water, transferred to the laboratory, and crushed within 4 hours of collection. Snails were crushed between two plates of a Chatillon DFM50 force gauge with an automated Chatillon LTMCM-6 stand. The mobile force plate was set at 2.54 cm/min crushing speed. The force in Newtons needed to crush the snail at the time of shell failure was recorded. To characterize shell size, we conducted a principal components analysis of log10-transformed linear shell measurements. To characterize shell shape, we performed a covariance PCA [35], which finds shape components that are separate from size. Covariance PCA analysis was conducted in ADE-4 [36], and PCA shape scores were obtained by an R program subroutine provided by A. Bellido. Using these PCA measures of size and shape, we conducted a stepwise multiple regression to assess whether size and shape explained significant variation in crushing resistance. Based on evidence that size explained considerable variation in crushing resistance (see Results), we next examined population-level variation in size-adjusted crushing resistance. We used analysis of covariance of crushing resistance under the following model: PCA size as a covariate, population as a main fixed effect, and an interaction term. We tested for homogeneity of slopes using the interaction term. Given a non-significant interaction term, we then conducted a two-way ANCOVA to assess whether there was a significant effect of population. We used the estimated marginal means as a measure of size-adjusted crushing resistance for each population in spatial autocorrelation analyses described next.

We used spatial autocorrelation analyses to examine both the independence of population estimates of crushing resistance and pigmentation, and whether defensive traits change in mosaic fashion at small spatial scales,. We used Moran's I to test for correlations among populations for two defensive traits (size-adjusted crushing resistance and frequency of pigmented shells), and whether these correlations change as a function of geographic and/or genetic distance. Under the spatial mosaic hypothesis, we predicted no positive autocorrelations of geographically-adjacent or genetically-similar populations. We estimated Moran's I using Passage [37]. Moran's I is a measure of autocorrelation, with positive autocorrelations indicating genetically-similar or geographically-proximate populations are similar in defensive traits [23,25]. We constructed plots of geographic and genetic distances against size-adjusted crushing resistance and percentages of pigmented snails (correlograms) using classes of geographic and genetic distances. We used an equal number of pairs of points for each distance class. We used GPS to determine latitude and longitude of each population and then calculated the surface distance between them in order to produce a geographic distance matrix of the Cuatro Ciénegas populations [38]. Pairwise genetic distances between populations were determined from a previously published paper on mtDNA cytochrome *b *sequence variation [26] using a distance matrix employing the number of pairwise differences.

### Abiotic and Biotic Influences on Load Strength and Pigmentation

To assess the potential influence of abiotic factors on shell strength, we measured temperature and conductivity in the 19 populations (Oakton hand-held conductivity meter Tampa, FL). Conductivity measures were temperature compensated. Above ground flow connects a few of the populations examined here, but the abiotic factors of each are influenced by large discharge from separate isothermal springs [7]. Therefore, the abiotic factors of the 19 populations should be fairly constant, and our measurements should be representative of the temperatures and conductivity populations experience during the entire year. We determined correlations between population means of putative defensive traits (size-adjusted load strength, frequency of pigmented shells) and both temperature and conductivity.

To estimate the relationship between frequency of molariforms and both size-adjusted crushing resistance and frequency of pigmented shells, the frequency of molariforms in ten populations was obtained from two sources. The frequency in Mojarral Oeste, North Tio Candido, Tio Candido, Los Remojos Negro, and Los Remojos Blanco was estimated in 2001 by Kloeppel [22], and was estimated in 2001 in Juan Santos and determined for Churince and Mojarral Este Alta in 2003 from samples reported in Hulsey *et al *[15]. Fish were considered molariform if they exhibited at least one molariform tooth. The correlation between these molariform frequencies and both size-adjusted crushing resistance and frequency of pigmented shells was then determined.

To address whether the presence or absence of *Nymphaea *is associated with variation in snail crushing resistance and pigmentation, we compared the crushing resistance (Analysis of Variance) and frequency of banded snails (Log likelihood tests) in three pairs of adjacent populations in the Rio Mesquites, Los Remojos, and Tio Candido. These paired populations are in very close proximity to one another (0.83 km, 0.05 km, and 0.48 km, respectively). In these paired populations, substrate coloration differed dramatically due to the presence or absence of *Nymphaea *beds. We also examined the phenotypic correlation between mean population estimates of the number of bands and crushing resistance. We conducted all statistical analyses in SPSS [39].

## Authors' contributions

All authors conceived the study and participated in its design and conducted field work. SGJ performed statistical analysis and drafted the manuscript. CDH and FJGL helped with revisions of the manuscript. All authors read and approved the final manuscript.
